# Sulfated Hyaluronan Derivatives Modulate TGF-β1:Receptor Complex Formation: Possible Consequences for TGF-β1 Signaling

**DOI:** 10.1038/s41598-017-01264-8

**Published:** 2017-04-26

**Authors:** Linda Koehler, Sergey Samsonov, Sandra Rother, Sarah Vogel, Sebastian Köhling, Stephanie Moeller, Matthias Schnabelrauch, Jörg Rademann, Ute Hempel, M. Teresa Pisabarro, Dieter Scharnweber, Vera Hintze

**Affiliations:** 1 Institute of Materials Science, Max Bergmann Center of Biomaterials, TU Dresden, Budapester Straße 27, 01069 Dresden, Germany; 2 Structural Bioinformatics, BIOTEC TU Dresden, Tatzberg 47-51, 01307 Dresden, Germany; 3 Medical Department, Institute of Physiological Chemistry, TU Dresden, Fiedlerstraße 42, 01307 Dresden, Germany; 4 Institute of Pharmacy, Freie Universität Berlin, Königin-Luise-Straße 2+4, 14195 Berlin, Germany; 5 Biomaterials Department, INNOVENT e.V., Prüssingstraße 27 B, 07745 Jena, Germany

## Abstract

Glycosaminoglycans are known to bind biological mediators thereby modulating their biological activity. Sulfated hyaluronans (sHA) were reported to strongly interact with transforming growth factor (TGF)-β1 leading to impaired bioactivity in fibroblasts. The underlying mechanism is not fully elucidated yet. Examining the interaction of all components of the TGF-β1:receptor complex with sHA by surface plasmon resonance, we could show that highly sulfated HA (sHA3) blocks binding of TGF-β1 to its TGF-β receptor-I (TβR-I) and -II (TβR-II). However, sequential addition of sHA3 to the TβR-II/TGF-β1 complex led to a significantly stronger recruitment of TβR-I compared to a complex lacking sHA3, indicating that the order of binding events is very important. Molecular modeling suggested a possible molecular mechanism in which sHA3 could potentially favor the association of TβR-I when added sequentially. For the first time bioactivity of TGF-β1 in conjunction with sHA was investigated at the receptor level. TβR-I and, furthermore, Smad2 phosphorylation were decreased in the presence of sHA3 indicating the formation of an inactive signaling complex. The results contribute to an improved understanding of the interference of sHA3 with TGF-β1:receptor complex formation and will help to further improve the design of functional biomaterials that interfere with TGF-β1-driven skin fibrosis.

## Introduction

Transforming growth factor (TGF)-β1 is a member of the TGF-β superfamily that consists of structurally and functionally related cytokines including three different forms of TGF-β, the bone morphogenetic proteins (BMPs), nodals, activins, inhibins and growth differentiation factors^1–3^. These cytokines are produced by diverse cell types, e.g. fibroblasts, endothelial cells as well as immune cells, and are known to regulate cell migration, adhesion, proliferation and differentiation^4, 5^. Members of the TGF-β superfamily signal across cell membranes in a distinctive manner by assembling heterotetrameric complexes of structurally related serine/threonine-kinase receptor pairs^6^. Among the TGF-β family, activins and nodals as well as BMPs promiscuously share type I and type II receptors whereas the TGF-βs interact with their receptors specifically^6–9^. TGF-β receptor type II (TβR-II) has high affinity for the dimeric TGF-β while TGF-β type I receptor (TβR-I) does not, despite the presence of a ligand binding domain^7, 10^. The different affinities of TβR-II and TβR-I to the ligand dictate a sequential order of complex assembly^11^ where TGF-β1 binds to TβR-II first and they both form a composite interface for the recruitment of TβR-I^7–9^. Upon binding of TGF-β, TβR-II trans-phosphorylates the low-affinity TβRI^6, 12, 13^. Activated type I receptors proceed to phosphorylate the cytoplasmic effector proteins Smad2 and Smad3 resulting in Smad4-containing heteromeric complexes of Smads 2 and 3, which are then translocated to the nucleus to regulate transcription of numerous target genes^2, 14–16^. In addition, TGF-β1 can activate specific non-Smad signaling pathways belonging to the mitogen-activated protein kinase pathways, including extracellular signal-regulated kinase 1/2 (Erk1/2)^17^. TGF-β1 is a key regulator of the production and remodeling of extracellular matrix (ECM) and plays a critical role in all phases of wound healing as it has a distinct influence on the regulation of multiple cellular responses. It is found at high levels in the wound microenvironment, where it promotes fibroblast chemotaxis, myofibroblast differentiation and induces fibroblasts to synthesize and contract extracellular matrix for wound contraction^1, 2^. Consistent overexpression of TGF-β1 during wound healing leads to an excessive accumulation of ECM proteins which clinically manifests in fibrotic skin disorders, like hypertrophic scarring, keloids and localized or systemic sclerosis^18–20^. TGF-β1 is known to interact with heparin and heparan sulfate (HS). Binding to these glycosaminoglycans (GAGs) potentiates TGF-β1 activity and prevents proteolytic degradation *in vitro* ^21, 22^. Hintze *et al*. demonstrated specific interactions between chemically sulfated hyaluronan HA (sHA) derivatives and TGF-β1 where the respective binding strength depended on the degree of HA sulfation and the highly sulfated HA (sHA3, three sulfate groups per repeating disaccharide unit of HA) exhibited the strongest interaction with TGF-β1^23^. In previous studies sHA derivatives have been shown to be a promising tool for investigating the structure-function relationship of GAGs in their interaction with biological mediators^23–28^. Compared to natively sulfated GAGs such as heparin they have defined properties regarding their monosaccharide composition and sulfation. Van der Smissen *et al*. investigated the consequences of TGF-β1 interaction with sHA derivatives on its bioactivity *in vitro* ^29^. They revealed an impaired Smad2/3 translocation to the nucleus in the presence of sHA3 in human dermal fibroblasts and proposed that sHA prevents interaction of TGF-β1 with TβR-I and -II. In the present study, the impact of sHA on the TGF-β1:receptor complex formation was investigated to explain the reduced TGF-β1 bioactivity. Using surface plasmon resonance (SPR) combined with molecular docking and molecular dynamics simulation techniques the complex interaction of TGF-β1 and both TGF-β receptors with sHA derivatives were examined for the first time. Moreover, the consequences of TGF-β1/GAG interaction and altered TGF-β1:receptor complex formation for TGF-β1-mediated TβR-I phosphorylation was examined using Western Blot analysis. In addition, TGF-β1-induced phosphorylation of Smad2 and Erk1/2 was evaluated.

## Results

### Characterization of Polymeric HA Derivatives

Low-, medium- and high-sulfated HA derivatives (sHA1, D.S. = 1.0; sHA2, D.S. = 1.8; sHA3, D.S. = 2.8) with reduced molecular weight (MW (LLS) of 26 kDa, 29 kDa and 21 kDa, respectively) were synthesized and characterized as described previously^24, 29^. As a non-sulfated reference material, a low molecular weight HA with a molecular weight of M_W_ (LLS) of about 48 kDa was prepared by controlled thermal degradation of native high molecular weight HA. ^13^C-NMR investigations of this degraded HA did not show any structural changes compared to native HA.

### Characterization of Tetrameric HA Derivatives

The persulfated and anomerically fixed HA degree of polymerization (dp) 4 was characterized by proton and carbon NMR-spectroscopy. The signals of the sugar ring showed the expected downfield shifts in the range of e.g. 0.46–0.61 ppm for the anomeric protons compared to the non-sulfated azide of HA dp4. The mass spectrometric analysis required a counter-ion exchange of all sulfate residues from sodium to tetraethylammonium (TEA) to reduce sulfate loss during the ionization process. The obtained ESI-MS-spectra of the per-sulfated azide of HA dp4 showed multiple charged species and different numbers of attached TEA counter-ions as described in Köhling *et al*.^30^. The average sulfate content of 80% of all mass signals that were detected and assigned amounted to a value of 8.8 sulfate groups per ion.

### SPR Analysis of TGF-β1 Binding to TβR-I and TβR-II after Pre-incubation with GAG Derivatives

**Figure 1:**
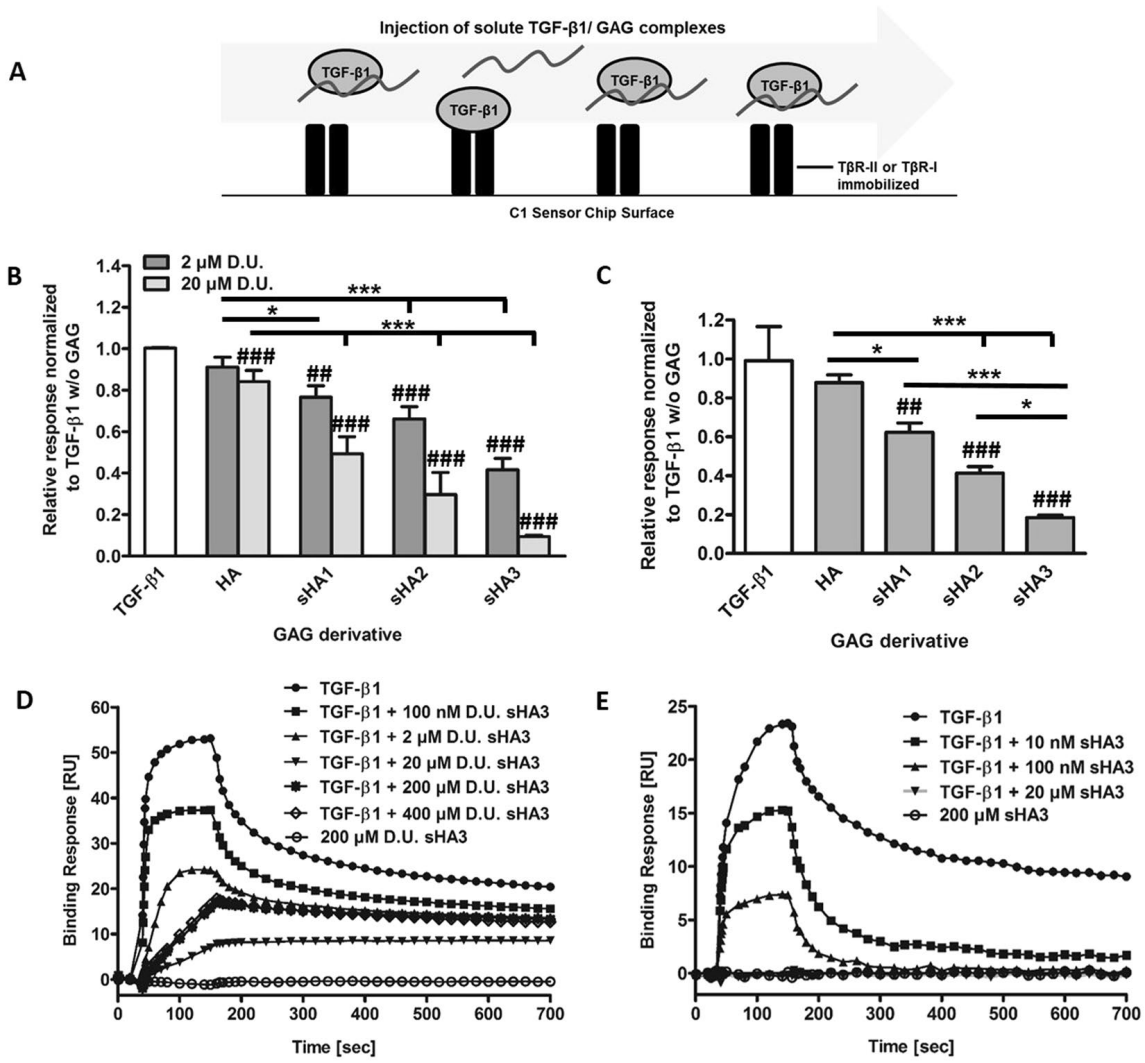
Binding of TGF-β1 to immobilized TβR-II and TβR-I after pre-incubation with different GAG derivatives. (**A**) Schematic drawings of the experimental set-up. (**B**) Relative binding of 40 nM TGF-β1 to TβR-II alone and after pre-incubation with 2 and 20 µM D.U. of HA, sHA1, sHA2 as well as sHA3. (**C**) Relative binding of 120 nM TGF-β1 to TβR-I alone and after pre-incubation with 100 nM D.U. of HA, sHA1, sHA2 as well as sHA3. (**D**) Sensorgrams for the binding of 40 nM TGF-β1 to TβR-II or (**E**) 120 nM TGF-β1 to TβR-I alone, after pre-incubation with 0.1, 2, 20 and 200 µM D.U. of sHA3 and for 200 µM D.U. of sHA3 without TGF-β1. For (**B**) and (**C**) values represent the mean ± SD of n = 3. One-way ANOVA: *p < 0.05; ***p < 0.001 vs. HA; ^##^p < 0.01; ^###^p < 0.001 vs. TGF-β1 only. For (**D**) and (**E**) one representative sensorgram out of three measurements is shown.

**Figure 2:**
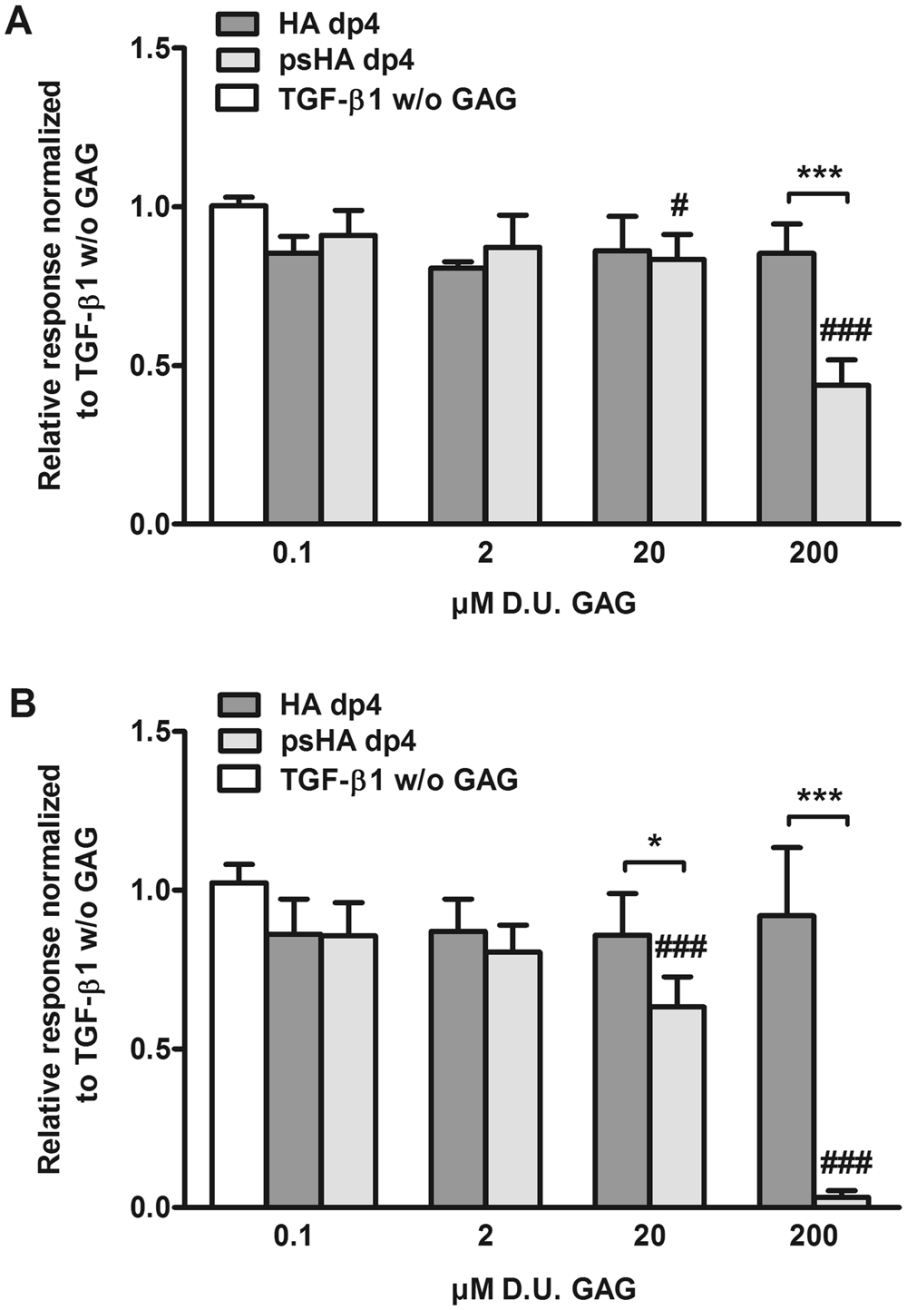
Binding of TGF-β1 to immobilized TβR-II and TβR-I after pre-incubation with tetrameric GAG derivatives. (**A**) Relative binding of 40 nM TGF-β1 to TβR-II alone and after pre-incubation with 0.1, 2, 20 and 200 µM D.U. of HA dp4 and psHA dp4. (**B**) Relative binding of 120 nM TGF-β1 to TβR-I alone and after pre-incubation with 0.1, 2, 20 and 200 µM D.U. of HA dp4 and psHA dp4. Values represent the mean ± SD of n = 3. Two-way ANOVA: *p < 0.05; ***p < 0.001 vs. respective treatment; ^#^p < 0.05; ^###^p < 0.001 vs. TGF-β1 only.

The impact of TGF-β1/GAG interaction on the TGF-β1:receptor complex formation was analyzed via SPR. Single TβRs were immobilized onto sensor chip surfaces and their interaction with TGF-β1 pre-incubated with different concentrations of HA derivatives was evaluated. While GAG derivatives alone did not bind to TβRs, increasing concentration and sulfation of the sHA derivatives led to a significantly decreased binding of TGF-β1 to TβR-II (Fig. 1B) and TβR-I (Fig. 1C). sHA3 had the most pronounced impact of all sHA derivatives studied. The binding response of TGF-β1 to TβR-II decreased with increasing concentrations of polymeric sHA3 but only up to a concentration of 20 µM related to the molecular weight of disaccharide units (D.U.). At concentrations above 20 µM D.U. the binding response increased again and ultimately reached a plateau at concentrations above 200 µM D.U. (Fig. 1D). At concentrations above 20 µM D.U. a change in the binding curves for TGF-β1 binding to TβR-II was detected, where the typical shape changed to a linear and monotonously rising one. This effect was also observed for TGF-β1 pre-incubated with sHA2 but not for sHA1 and HA (Supplementary Figure S1). In contrast to the findings for TβR-II, binding of TGF-β1 to TβR-I was completely suppressed in the presence of 20 µM D.U. sHA3 (Fig. 1E). To determine the minimal binding sequence required to interfere with the interaction of TGF-β1 and its receptors HA dp4 and persulfated HA (psHA) dp4 were used. psHA dp4 inhibited binding of TGF-β1 to the TβRs as well (Fig. 2). As for the polymeric HA derivatives the effect on TGF-β1 binding to TβR-I was more pronounced compared to TβR-II. In contrast, HA dp4 only slightly inhibited the binding of TGF-β1 to its receptors and did not exhibit a concentration-dependent effect.

### Sequential SPR Analysis of TGF-β1:Receptor Complex Formation in the Presence of Different GAG Derivatives

**Figure 3:**
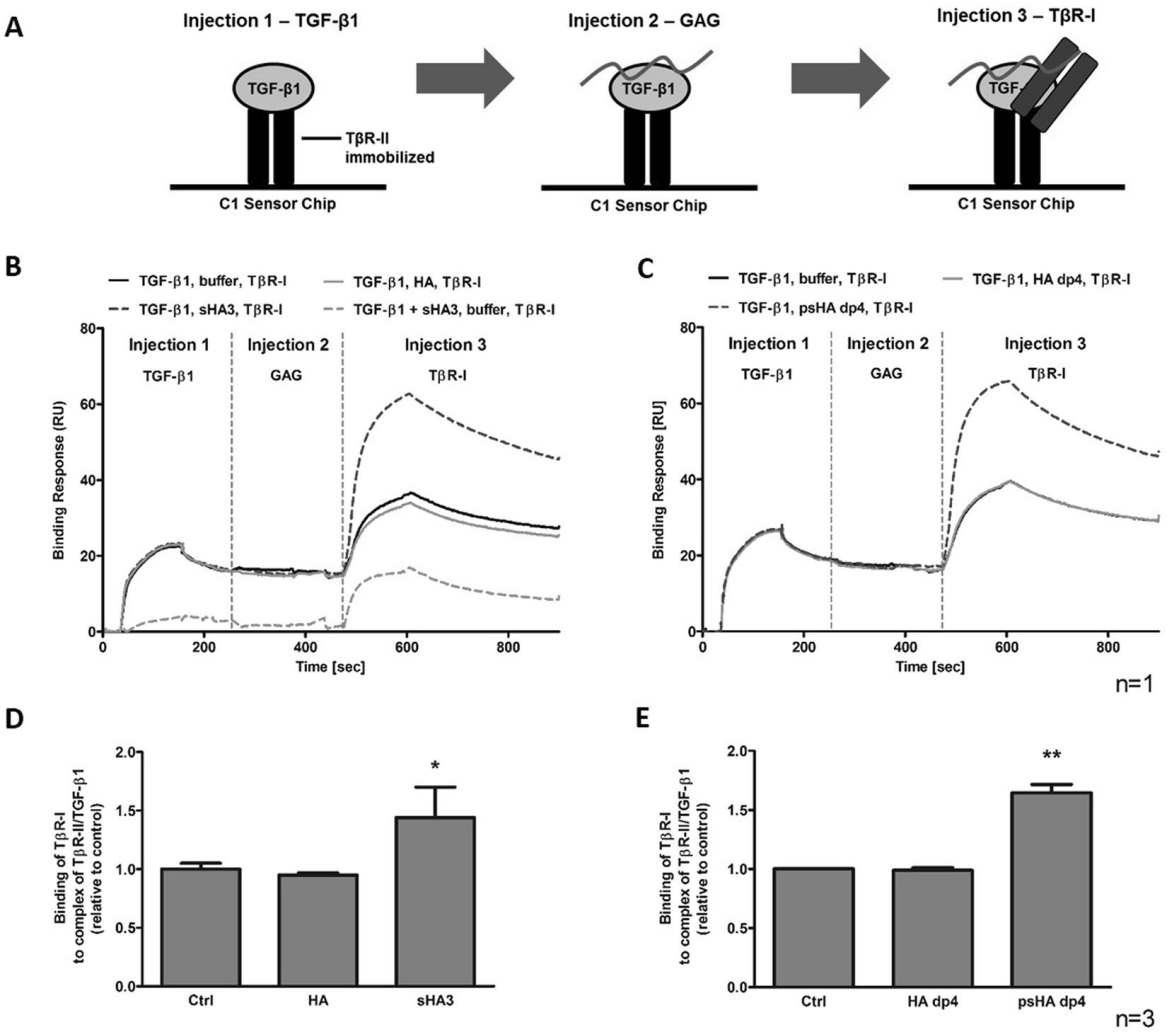
Sequential TGF-β1:receptor complex formation in the presence of different HA derivatives. (**A**) Schematic drawing of the experimental set-up showing that TβR-II was immobilized on a C1 Sensor Chip and all other components were injected in the order: TGF-β1, GAG or buffer, TβR-I. Binding of TβR-I to TβR-II/TGF-β1 in the presence of (**B**) polymeric HA and sHA3 or (**C**) HA dp4 and psHA dp4. The sensorgrams display one representative experiment out of at least three independent measurements. Binding levels for the association of TβR-I to the complex of TβR-II/TGF-β1 including the indicated (**D**) polymeric or (**E**) tetrameric GAGs were ranked. Values represent the mean ± SD of n = 3. One-way ANOVA: *p < 0.05; **p < 0.01 vs. control or HA.

The impact of HA derivatives on the sequential formation of the TGF-β1:receptor complex containing both receptors was investigated. TβR-II was immobilized on a sensor chip surface and the other components were injected sequentially in the following order: 40 nM TGF-β1, 100 µM D.U. GAG and 40 nM TβR-I. The recruitment of TβR-I to the complex of TβR-II/TGF-β1/sHA3 was significantly enhanced, compared to complexes with no GAG derivative (TGF-β1, buffer, TβR-I, Fig. 3B and D). A similar effect occured for psHA dp4, when injected in the same sequential order (Fig. 3C and E). When pre-formed TGF-β1/sHA3 complexes where injected first followed by the injection of TβR-I, recruitment of TβR-I to the complex was enhanced as well, although the binding of TGF-β1/sHA3 pre-formed complexes to TβR-II was lower compared to TGF-β1 alone (Fig. 3B). For both polymeric HA and HA dp4 there was no enhanced TβR-I recruitment.

### Molecular Modeling of the TGF-β1/TβR-I/TβR-II/GAG System

**Figure 4:**
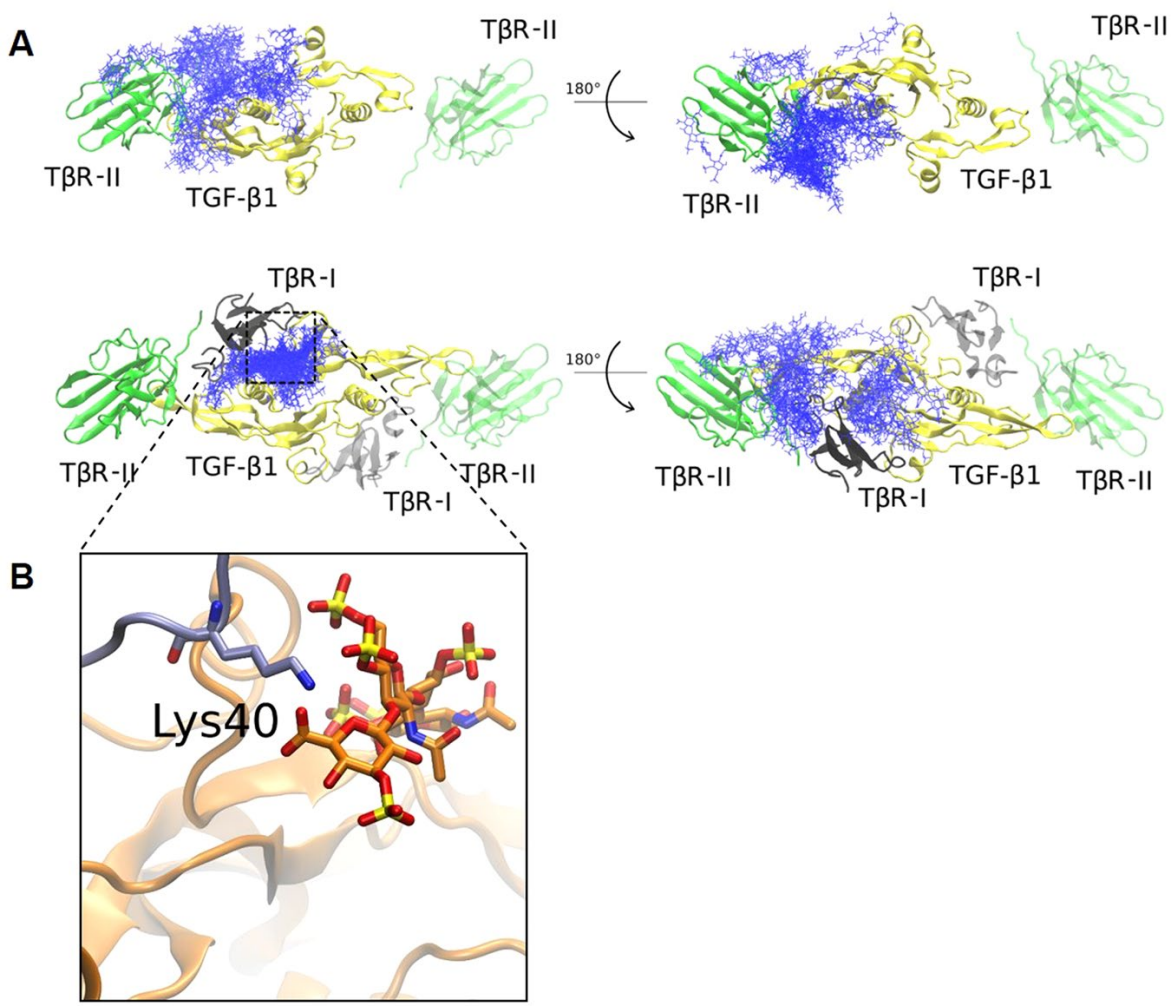
Molecular Modeling of the TGF-β1/TβR-I/TβR-II/GAG System. (**A**) Docking results for HA463′ dp4 (top 50 solutions, in blue sticks) to TGF-β1/TβR-II (top) and TGF-β1/TβR-II/TβR-I (bottom). TGF-β1 is shown in yellow, TβR-II in green and TβR-I in grey. The part of the system not used for docking calculations (TβR-I and TβR-II on the right side of TGF-β1) is depicted with transparency. (**B**) Zoomed view of the residue Lys40 of TβR-I (in thick sticks, carbons in cyan) that interacts with the negatively charged groups of HA463′ dp4 (in thick sticks, carbons in orange). Protein is shown in cartoon.

Docking calculations and MD simulations were carried out to investigate the molecular mechanism of enhanced TβR-I recruitment to the complex of TβR-II/TGF-β1/sHA3 found in SPR measurements. When docking GAGs to TGF-β1/TβR-II, we found poorly clustered and broadly distributed docking solutions (Fig. 4A, upper panel). Some of them spatially overlapped with the TβR-I binding site and for this reason could not be used to explain the experimental results obtained for TβR-I recruitment. Binding poses corresponding to the obtained loose clusters non-overlapping with the TβR-I binding site were extracted for further analysis using an MD approach and free energy calculations. GAGs in these binding poses did not promote the association of TGF-β1/TβR-II/GAG with TβR-I. However, when docked to TGF-β1/TβR-II/TβR-I, a clear GAG binding pose could be predicted (Fig. 4A, down panel). Analyzing docking solutions obtained for the TGF-β1/TβR-II/GAG complex, we found the same binding pose within the top 50 solutions, though, in this case, it was not representative in terms of clustering. MD analysis of this binding pose showed that it energetically favors the association of TGF-β1/TβR-II/GAG with TβR-I in case of HA463′ but not in case of HA, and that the corresponding interactions are electrostatically driven (Table 1). Analysis of the per residue impact to TGF-β1/TβR-II/TβR-I/GAG complex association showed that residue Lys40 of TβR-I (Fig. 4B) plays a key role for the interactions with a pre-bound GAG through its carboxyl and sulfate groups.

**Table 1:** MM-PBSA analysis of TβR-I binding to TGF-β1/TβR-II in the presence and absence of GAGs.

System	ΔG, kcal/mol	^**^ΔG_elect_, kcal/mol	ΔG_K40_, kcal/mol
TGF-β1/TβR-II/TβR-I	−68.9 ± 12.5	−301.3 ± 28.9	0.2
TGF-β1/TβR-II/HA_dp4_/TβR-I^*^	−63.3 ± 12.9	−415.9 ± 35.4	0.0
TGF-β1/TβR-II/HA_dp8_/TβR-I	−83.1 ± 9.6	−435.3 ± 39.1	−3.3
TGF-β1/TβR-II/HA463′_dp4_/TβR-I	−83.1 ± 8.4	−420.0 ± 48.1	−2.7
TGF-β1/TβR-II/HA463′_dp8_/TβR-I	−84.9 ± 12.7	−451.1 ± 76.7	−2.9

*GAG did not behave stable in the MD and dissociated; **Electrostatic component.

### Influence of GAG Derivatives on TGF-β1-mediated TβR-I and Smad2 Phosphorylation

**Figure 5:**
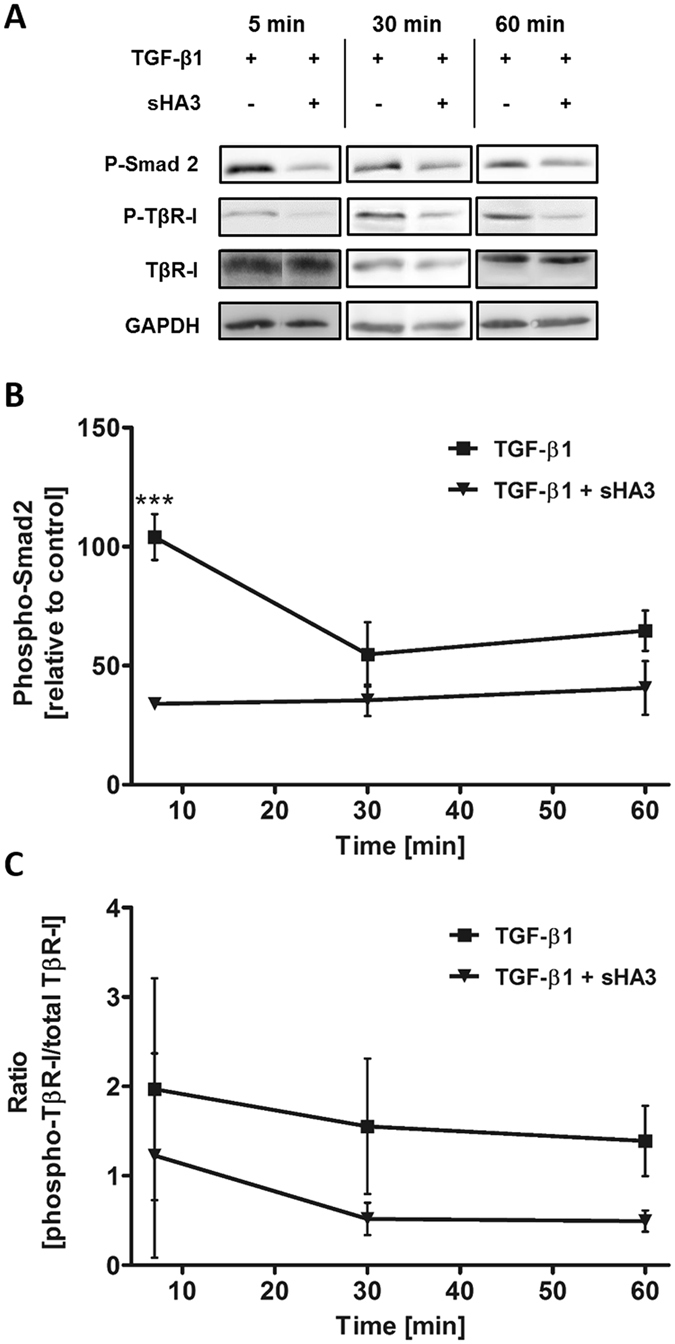
Influence of sHA3 on the TGF-β1-mediated TβR-I and Smad2 phosphorylation. Hs27 fibroblast cells were stimulated with 10 ng/ml TGF-β1 (0.4 nM) alone or pre-formed complexes of TGF-β1 and 100 µg/ml sHA3 (130 µM D.U.) for the indicated time points. Cells were lysed and applied to Western Blot analyses using specific anti-phospho-Smad2, anti-phospho-TβR-I and anti-GAPDH antibodies. Membranes were reblotted afterwards with anti-TβR-I antibody. For every time point a representative blot is shown (**A**). The time course of TGF-β1-mediated phosphorylation in the presence or absence of sHA3 is plotted for Smad2 phosphorylation (**B**) and TβR-I phosphorylation relative to unstimulated cells (**C**). Two-way ANOVA: ***p < 0.001 vs. TGF-β1 only.

Previous experiments showed that TGF-β1:receptor complex formation was altered in the presence of sHA3. Western Blot analyses were performed to determine the consequences of this change on the phosphorylation of TβR-I and the second messenger molecules Smad2 and Erk1/2. Hs27 fibroblast cells treated with 10 ng/ml TGF-β1 displayed a strong Smad2 phosphorylation signal compared to the unstimulated control. Stimulation with pre-formed TGF-β1/sHA3 complexes led to a decrease in TGF-β1-mediated Smad2 phosphorylation at all time points investigated, which was significant after 5 min (Fig. 5A and B). TβR-I was already phosphorylated in untreated cells, but was enhanced in the presence of 10 ng/ml TGF-β1. Treatment with TGF-β1/sHA3 complexes reduced TβR-I phosphorylation compared to the total amount of TβR-I, although the differences are not significant (Fig. 5A and C). Regarding the phosphorylation of Erk1/2 no influence of sHA3 was observed (Supplementary Figure S2).

## Discussion

Chemically sulfated HA derivatives are promising candidates for engineering functional biomaterials since their sulfate groups modulate binding and bioactivity of growth factors, which in turn can influence healing processes. Previous studies by van der Smissen *et al*. showed sHA derivatives to impair TGF-β1 downstream signaling by reducing Smad2/3 translocation to the nucleus. *In silico* docking experiments suggested that occupation of the receptor binding sites on TGF-β1 by sHA might be responsible for this effect^29^. The aim of the present study was twofold: to achieve better understanding of sHAs’ impact on TGF-β1:receptor complex formation by combining SPR analysis and computational approaches and to determine the consequences of an altered TGF-β1:receptor complex formation on the receptor level by investigating TβR-I phosphorylation in addition to phosphorylation of the TβR-I regulated effector protein Smad2^15^. Studies by Hintze *et al*. revealed that TGF-β1 interacts with sHA derivatives in a sulfation- dependent manner, demonstrating the strongest interaction with highly sulfated sHA3^23^. In the present study, pre-incubation of TGF-β1 with sHA derivatives blocked the binding of TGF-β1 in particular to TβR-I, but also to TβR-II. sHA3 exhibited the strongest inhibitory effect and completely blocked binding of TGF-β1 to TβR-I at 20 µM D.U., while binding to TβR-II was not fully constrained even at concentrations above 200 µM D.U. sHA3. This is in line with the low affinity of TβR-I for the ligand alone (K_D_ = 70 µM), while the affinity of TβR-II is considerably higher (K_D_ = 190 nM)^31^. In our experiments, the lower affinity of TβR-I was reflected by the fact that a three times higher concentration of TGF-β1 was needed to receive a binding response for TβR-I comparable to TβR-II. However, inhibition of binding to TβR-I might not be as relevant as to TβR-II, since interactions between TGF-β1 and TβR-I *in vivo* are barely detectable^31^. The SPR data are consistent with observations from previous studies including TGF-β1 and sHA derivatives predicting a sulfation-dependent occupation in particular of the TβR-I binding site of TGF-β1 by tetrameric sHA derivatives^29^. sHA preferred the TβR-I binding site on TGF-β1 due to a more favorable distribution of positively charged residues, which are important for GAG recognition. Longer GAG molecules bound to the TβR-I site might impair binding of the ligand to the TβR-II site as well, since the binding sites are in close proximity and thus sterical hindrance could occur. In binding experiments of the present study psHA dp4 exhibited a strong inhibitory effect on the association of TGF-β1 and TβR-II as well, though weaker than the effect on TGF-β1 binding to TβR-I. The interference of GAGs with TβR-I binding was dependent on GAG net charge indicating the importance of electrostatics in these interactions. Interestingly, an increase in binding response for binding of TGF-β1 to TβR-II could be observed in the presence of sHA3 at concentrations above 20 µM D.U. compared to lower sHA3 concentrations. Moreover, the curvature in the sensorgrams was different for binding of TGF-β1 to TβR-II in the absence of sHA3. The slope of the binding curves was more linear and binding to TβR-II did not further decrease. TGF-β1 and sHA3 might have formed complexes that were able to bind TβR-II even though the binding strength was weaker than for TGF-β1 alone. This is consistent with previous docking experiments revealing that GAGs only partially impair TGF-β1 binding to TβR-II^29^. As TGF-β1:receptor complex formation is described as an ordered-sequential assembly mode sequential SPR experiments were performed. *In vivo*, the different affinities of TβR-II and TβR-I to the ligand dictate a sequential order of complex assembly: TGF-β1 binds TβR-II first, followed by the recruitment of TβR-I^8, 9^. Interestingly, a significantly stronger interaction of TβR-I with the complexes of TβR-II/TGF-β1/sHA3 and TβR-II/TGF-β1/psHA dp4 was observed, which did not occur in the presence of HA or HA dp4. In contrast to our findings with pre-formed TGF-β1/sHA complexes, binding of TβR-I to TβR-II/TGF-β1/sHA3 was not blocked by sHA3, but the interaction was enhanced compared to TβR-I binding to TβR-II/TGF-β1. Thus, when TGF-β1 and sHA3 were injected consecutively, TGF-β1 bound to TβR-II still had free binding sites for sHA3. A similar effect was observed with psHA dp4, even though the recruitment was lower compared to polymeric sHA3. When TGF-β1 and sHA3 were injected as pre-formed complexes over immobilized TβR-II in the sequential experiments TβR-I recruitment to the complex of TβR-II/TGF-β1/sHA3 was enhanced as well. This further indicates that TGF-β1/sHA3 complexes with sHA3 concentrations above 20 µM D.U. were able to bind TβR-II. In general, the binding levels for this interaction were low, due to a lower immobilization level of TβR-II. However, a stable binding of TGF-β1/sHA3 complexes to TβR-II was already shown in Fig. 1D. Recruitment of TβR-I was still enhanced, but due to the lower binding response of pre-formed TGF-β1/sHA3 complexes the effect was less pronounced compared to consecutively injected TGF-β1 and sHA3 showing a higher binding signal. Molecular modeling supported the sequential SPR findings and provide further insights into the potential molecular mechanism underlying the effect of GAG recognition on the function of TGF-β1:receptor:GAG system. There were different binding poses for GAGs depending on the presence or absence of the receptors. If the TβRs were not included in the docking experiment, GAGs interfered with the TGF-β receptor binding sites. However, inclusion of the receptors led to alternative putative binding poses for the GAGs. In the presence of TβR-II alone these were broadly distributed, but if TβR-I was included a clear binding pose emerged. Furthermore, sHA oligosaccharides led to a more stable interaction with TβR-I than non-sulfated HA oligosaccharides if pre-bound to TβR-II/TGF-β1. This could be explained by electrostatic interactions of the GAGs carboxyl and sulfate groups with Lys40 of TβR-I. The interaction was stronger for GAGs with a high sulfation degree (3 vs. 0) and chain length (dp8 vs dp4). sHA showed no clear binding pose in the complex with TβR-II/TGF-β1 in the absence of TβR-I. This may indicate multiple binding sites of the GAG on TGF-β1 that allow for TβR-I association to the complex. Lyon *et al*. proposed a binding model in which GAG binding occurs at two distinct sites on the TGF-β1 dimer, which was confirmed by Lee *et al*., postulating that the second binding site is located on the face opposite of the primary binding site^21, 32^. Even if TβR-I association was increased the complex that formed in the presence of sHA3 was inactive as Western Blot experiments revealed an impaired Smad2 phosphorylation in the presence of sHA3. Immunofluorescence staining of Smad2/3 in primary fibroblasts incubated with sHA3 showed a reduced translocation of the second messengers to the nucleus^29^ which is in line with our data showing a reduced Smad2 phosphorylation in a fibroblast cell line. In addition, a trend for a decreased TβR-I phosphorylation in the presence of sHA3 was observed as well. In the TGF-β1 pathway, Smad2 and Smad3 are receptor-regulated effector proteins, specifically phosphorylated by activated TβR-I^15^. A reduced Smad2 phosphorylation would thereby be coherently explained by a previously reduced receptor phosphorylation due to altered TGF-β1:receptor formation. For the phosphorylation of Erk1/2, however, no impact of GAGs could be observed. This might be due to Erk1/2 being regulated by a multitude of other factors and not being activated by the Smad pathway^33, 34^. Binding studies with the sulfated polysaccharide fucoidan showed an inhibitory effect on the binding of TGF-β1 to TβR-II resulting in a decreased Smad2 phosphorylation as well^35^. However, in this study a three times lower concentration of sulfated polysaccharide was used compared to the present study, and TβR-I was not included in the binding studies. In contrast to this, Lyon *et al*. found that heparin and highly sulfated liver HS potentiated the activity of TGF-β1 in rat kidney fibroblasts, indicating that there is no competition between GAG binding and receptor binding but some form of cooperativity. Nevertheless, the effect was only observed in the presence of α_2_-macroglobulin (α_2_M) and therefore ascribed to an antagonistic effect of heparin and HS rescuing the inactivation of TGF-β1 by α_2_M, rather than to the modulation of TGF-β1:receptor interaction^21, 36^. Chemically sulfated dextrans were also found to potentiate the biological activity of TGF-β1, but this was explained by a mere protection of TGF-β1 from proteolysis upon complex formation with dextrans^37^. It should also be taken into account that conclusions are based on cell experiments with mink lung epithelial cells. We recognize that our study has potential limitations. The receptors used for SPR experiments are Fc-fusion proteins. As these receptor chimera are dimeric avidity through bivalent interactions cannot be excluded in sequential SPR experiments and TβR-II and TβR-I might form a ligand-independent complex in addition to complexes including TGF-β1. The enhanced TβR-I recruitment, however, is ligand-dependent as sHA derivatives do not bind to the receptors. Another potential limiting factor may be the fact that native TGF-β receptors are transmembrane receptors with a cytoplasmic tail involved in large functional complexes. Our study only uses the ECDs of the receptors in a simplified *in vitro* model, allowing only for a part of the complex interplay of the components *in vivo*. The present study reveals the underlying mechanism of reduced TGF-β1 bioactivity in fibroblast cells in the presence of sHA3. The findings have a strong impact on the elucidation of the mode of action of sHA derivatives, showing that they affect the association of TGF-β1 with both TGF-β receptors and that the order of binding events is highly important. By interacting with TGF-β1 GAGs subsequently alter TGF-β1:receptor complex formation either by blocking the interaction of TGF-β1 with its receptor or by partially forming a complex that does not activate the Smad signaling pathway. Together with van der Smissen *et al*. our data suggest that sHA derivatives are promising candidates for biomaterials as their inhibitory effect on TGF-β1 bioactivity might be useful to locally interfere with TGF-β1 driven skin fibrosis. Whether our *in vitro* findings can be translated *in vivo* needs to be evaluated extensively in future work.

## Methods

### Materials

Hyaluronan (from Streptococcus, MW = 1.1 × 106 g mol-1) was obtained from Aqua Biochem (Dessau, Germany) and sulfur trioxide/dimethylformamide complex (SO_3_–DMF, purum, ≥97%, active SO_3_ ≥ 48%) as well as sulfur trioxide/pyridine complex (SO_3_–pyridine, pract.; ≥45% SO_3_) from Fluka Chemie (Buchs, Switzerland). Biochemical agents were purchased from Sigma-Aldrich (Schnelldorf, Germany). Recombinant human TGF-β1 (240-B-010/CF) as well as recombinant human TGF-β receptor II/Fc Chimera (341-BR-050/CF) were obtained from R&D Systems (Wiesbaden-Nordenstadt, Germany) and recombinant human TGF-β receptor I/Fc Chimera (10459-H03H) from Hoelzel Diagnostika GmbH (Köln, Germany). For SPR measurements the Series S Sensor Chip C1, the Amine Coupling Kit and HBS-EP (10x) from GE Healthcare Europe GmbH (Freiburg, Germany) were used.

### Preparation of Polymeric HA Derivatives

Low molecular weight HA (LMW-HA) was produced via thermal degradation of native high molecular HA as described previously in Kunze *et al*.^38^ Low-, medium- and high-sulfated HA derivatives (sHA1, sHA2, sHA3) were synthesized and analytically characterized as described in Hintze *et al*.^23, 24^. Analytical data of the prepared HA derivatives (LMW-HA, sHA1, sHA2, sHA3) are summarized in Table 2.

**Table 2:** Characteristics of synthesized polymeric HA derivatives.

Sample	LMW-HA	sHA1	sHA2	sHA3
D.S.^a^	—	1.0	1.8	2.9
M_n_ [g/mol]^b^	28.250 (81.145)	18.260 (46.935)	14.980 (34.335)	11.680 (28.885)
M_w_ [g/mol]^c^	48.255 (187.010)	26.610 (102.730)	29.040 (72.520)	20 950 (47.720)
PD^d^	2.3	2.2	2.1	1.7

^a^D.S.: degree of sulfation, average number of sulfate groups per disaccharide unit of GAG. ^b^M_n_: number-average molecular weight, determined by Laser Light Scattering (LLS) detection and Refraction (RI) detection (in brackets). ^c^M_w_: weight-average molecular weight, determined by LLS detection and RI detection (in brackets). ^d^PD: polydispersity index based on the values calculated from RI detection.

### Preparation of Tetrameric HA Derivatives

Non-sulfated HA tetrasaccharide (degree of polymerization (dp) 4) was produced via enzymatic digestion of native HA with bovine testes hyaluronidase. Persulfated HA dp4 was synthesized after fixation of the anomeric configuration of the reducing end as azide with 2-chloro-1,3-dimethyl-imidazolinium chloride and sodium azide in the presence of *N*-methylmorpholine as base. The hydroxyl groups of the corresponding anomeric azide were chemically sulfated using sulfur trioxide pyridine complex as sulfating agent. After purification by dialysis, HA dp4 was obtained as nona-sulfate sodium salt (psHA, dp4) as described in Köhling *et al*.^30^.

### Immobilization of TβR-II and TβR-I on Sensor Chips

For interaction analysis of growth factor and receptors in the presence of GAGs, a BIACORE T100 instrument (GE Healthcare) was used. TβR-II and TβR-I, respectively, were immobilized on the surface of a Series S Sensor Chip C1 at 25 °C using the amine coupling reaction as described by the manufacturer resulting in an average of 200 RU TβR-II and 130 RU TβR-I immobilized to the chip surface using a concentration of 100 µg/ml. As a reference one flow cell was activated and directly deactivated without immobilizing the receptors. HBS-EP (0.01 M HEPES (pH 7.4), 0.15 M NaCl, 3 mM EDTA, 0.05% surfactant P20) was used as running buffer. Prior to interaction analysis, the chip surface with immobilized receptors was blocked with three injections of 1% (w/v) BSA, 5% (w/v) sucrose in HBS-EP (3 × 700 s at 30 µl/min).

### SPR Analysis of TGF-β1 Binding to TβR-I and TβR-II in the Presence of GAG Derivatives

All interaction studies were performed at 37 °C. GAG samples were diluted in HBS-EP. For interaction studies with TβR-II 100 nM-200 µM of the respective GAG derivative related to the molecular weight of disaccharide units (D.U.) were pre-incubated for 60 min at room temperature (RT) with 40 nM TGF-β1 in HBS-EP. For the analysis of binding to TβR-I 100 nM D.U. GAG were pre-incubated for 60 min at RT with 120 nM TGF-β1 in HBS-EP. After three start up injections with running buffer, pre-formed TGF-β1/GAG complexes or TGF-β1 alone were injected for 120 s at 30 µl/min and binding levels were recorded 10 s before injection stop. Additionally, 200 µM D.U. GAG alone were injected over the immobilized TβRs as a control. The injection was followed by a 10 min dissociation phase in running buffer at a flow rate of 30 µl/min. The sensor chip surface was regenerated after each sample injection with 20 mM HCl for 2 min at a flow rate of 5 µl/min^39^. The baseline was allowed to stabilize for 1000 s with running buffer prior to injection of the next sample. Data represent the mean of three independent measurements.

### Sequential SPR Analysis of TGF-β1:Receptor Complex Formation in the Presence of Different GAG Derivatives

TβR-II was immobilized on the sensor chip surface and three consecutive injections of TGF-β1, GAG derivatives and TβR-I were performed in each running cycle. 40 nM TGF-β1 were injected for 120 s at 30 µl/min with a dissociation phase of 30 s in running buffer. Afterwards 100 µM D.U. of the respective GAG derivative were injected under the same conditions followed by the injection of 40 nM TβR-I with a dissociation time of 300 s. Additionally, 40 nM TGF-β1 and 100 µM D.U. of the respective GAG derivative were pre-incubated for 1 h at RT. The pre- formed growth factor/GAG complexes were injected over immobilized TβR-II, followed by a buffer injection and the injection of TβR-I. The sensor chip surface was regenerated after each running cycle with a 30 µl pulse of 5 M NaCl in 30 mM NaOH followed by two injections of 20 mM HCl for 120 s at a flow rate of 5 µl/min. Data were double referenced by the response of the reference surface and the response of HBS-EP buffer alone relative to a baseline report point. Binding parameters were evaluated using the BIACORE T100 evaluation software 2.03.

### Molecular Docking

Autodock 3 (AD3)^40^ was used for docking GAG molecules to TGF-β1/TβR-II and TGF-β1/TβR-II/TβR-I complexes. The protein and receptor coordinates were obtained from their experimental crystal structure at the Brookhaven Protein Databank (PDB ID: 3KFD, 2.99 Å). HA (-GlcU-GlcNAc-)_*n*_ and HA463′ (-GlcU3S-GlcNAc4S6S-)_*n*_ of length dp4 and dp8 were left completely flexible in the docking runs. An atomic grid with the 0.375 Å spacing was used. 100 independent runs of the Lamarckian genetic algorithm with an initial population size of 300 and a termination condition of 10^5^ generations or 9995·10^5^ energy evaluations were carried out. The 50 top docking solutions were clustered using the DBSCAN algorithm^41^.

### Molecular Dynamics

The TGF-β1/TβR-I/TβR-II complex and the complexes with GAGs obtained by docking were simulated with the AMBER 11.0 package^42^ using ff99SB force field parameters for the protein molecule and GLYCAM06 for GAG molecules. These complexes were solvated with TIP3P water molecules in an octahedral periodic box with a minimal distance to the periodic box border of 6 Å and neutralized by counterions. The molecular dynamics (MD) simulations were run as described previously^43^ with MD productive runs of 10 ns. MM-PBSA free energy calculations of protein-GAG binding and MM-GBSA (igb = 2) per residue energy decomposition were done for 100 frames evenly distributed in each MD trajectory.

### Cell Culture and Western Blot Analysis of TβR-I Phosphorylation

Hs27 fibroblast cells (ATCC-CRL-1634, human foreskin fibroblast cell line) were cultivated in complete medium (Dulbecco’s Modified Eagle’s Medium) with 10% fetal calf serum, 1,5 g/l NaHCO_3_, 1% Penicillin/Streptomycin, 100 mM sodium pyruvate and 4 mM L-glutamine (Biochrom AG, Berlin, Germany) at 37 °C in a humidified atmosphere with 5% CO_2_. For experiments cells were seeded at a density of 13,000 cells/cm^2^ in T75 cell culture flasks (Sarstedt, Nümbrecht, Germany), grown to 80% confluency and serum-starved for 24 h. Cells were stimulated with 10 ng/ml (0.4 nM) TGF-β1 alone or with mixtures of TGF-β1 and 100 µg/ml HA (220 µM D.U.) or sHA3 (130 µM D.U.), pre-incubated for 2 h at 4 °C, for 5 min, 30 min and 60 min. Cells were washed with PBS and incubated in lysis buffer (50 mM Tris-HCl pH 7.4, 150 mM NaCl, 1% Nonidet-P40, 1 mM EDTA, 0.1% (v/v) SDS, 2 mM PMSF (AppliChem, Darmstadt, Germany), 0.1 mM aprotinin, 0.1 mM Na_3_VO_4_ and 5 mM NaF) at 4 °C for 10 min. Lysates were centrifuged at 13,000 × g for 30 min at 4 °C. Equal amounts of protein (20 µg) were subjected to 12% SDS-polyacrylamide gels and subsequently transferred to nitrocellulose membranes (GE Healthcare, Freiburg Germany). The membranes were incubated with 5% (w/v) BSA in 25 mM Tris-buffered saline, pH 8/0.5% (v/v) Tween-20 (TBST) or 5% (w/v) dry milk in TBST at RT for 2 h and primary antibody in TBST containing 5% BSA at 4 °C overnight. The following primary antibodies were used: rabbit-anti-human TGF beta Receptor I (Phospho-Ser165) pAB (MBS859620, My Bio Source via Biozol Diagnostica Vertrieb GmbH, Eching, Germany), rabbit-anti-human TGF beta Receptor I pAB (PA5–14959, Thermo Scientific, Schwerte Germany), rabbit-anti-human phospho-Smad2 (Ser465/467) pAB (3101, Cell Signaling Technology (CST) via New England Biolabs, Frankfurt/Main, Germany), rabbit-anti-human phospho-p44/42 MAPK (Erk1/2) (Thr202/Tyr204) pAB (9101, CST) and mouse-anti-human GAPDH mAB (CB1001, Calbiochem via Merck Millipore, Darmstadt, Germany). All primary antibodies were diluted 1:1000. Horseradish peroxidase (HRP)-conjugated secondary anti-rabbit-IgG and anti-mouse-IgG (CST) in TBST containing 5% dry milk at a dilution of 1:2000 were used as secondary antibodies. Immune complexes were detected using Immobilon Western Chemiluminescent HRP Substrate (Merck Millipore) and visualized by enhanced chemiluminescence detection using a CCD camera system (MF-ChemiBIS1.6 via Biostep Jahnsdorf, Germany). For re-blotting the membrane was incubated in stripping buffer (Thermo Scientific) at RT for 20 min. Afterwards the immune steps were repeated starting with dry milk incubation. Band signals were evaluated densitometrically using ImageQuantTM 5.1 software (GE Healthcare).

### Statistical Analysis

All experiments were performed in triplicate and results are presented as mean ± standard deviation (SD) for SPR results and mean ± standard error of the mean (SEM) for Western Blot results. One-way ANOVA or two-way ANOVA with Bonferroni post-test were applied. P values < 0.05 were considered statistically significant.

## Electronic supplementary material

Supplementary Information
