# Supplementary material for: Sulfated Hyaluronan Derivatives Modulate TGF-β1:Receptor Complex Formation: Possible Consequences for TGF-β1 Signaling
Source: Sci Rep. 2017 Apr 26;7:1210. doi: 10.1038/s41598-017-01264-8 (PMC5430790; doi:10.1038/s41598-017-01264-8)
Supplement: Supplementary file 1 — Supplementary Information [file 41598_2017_1264_MOESM1_ESM.pdf]

Supplementary Information

For Sci Rep SREP-16-41359B

Sulfated Hyaluronan Derivatives Modulate TGF-β1:Receptor Complex Formation: Possible Consequences for TGF- β1 Signaling

Linda Koehler, Sergey Samsonov, Sandra Rother, Sarah Vogel, Sebastian Köhling, Stephanie Moeller, Matthias Schnabelrauch, Jörg Rademann, Ute Hempel, M. Teresa Pisabarro, Dieter Scharnweber, Vera Hintze*

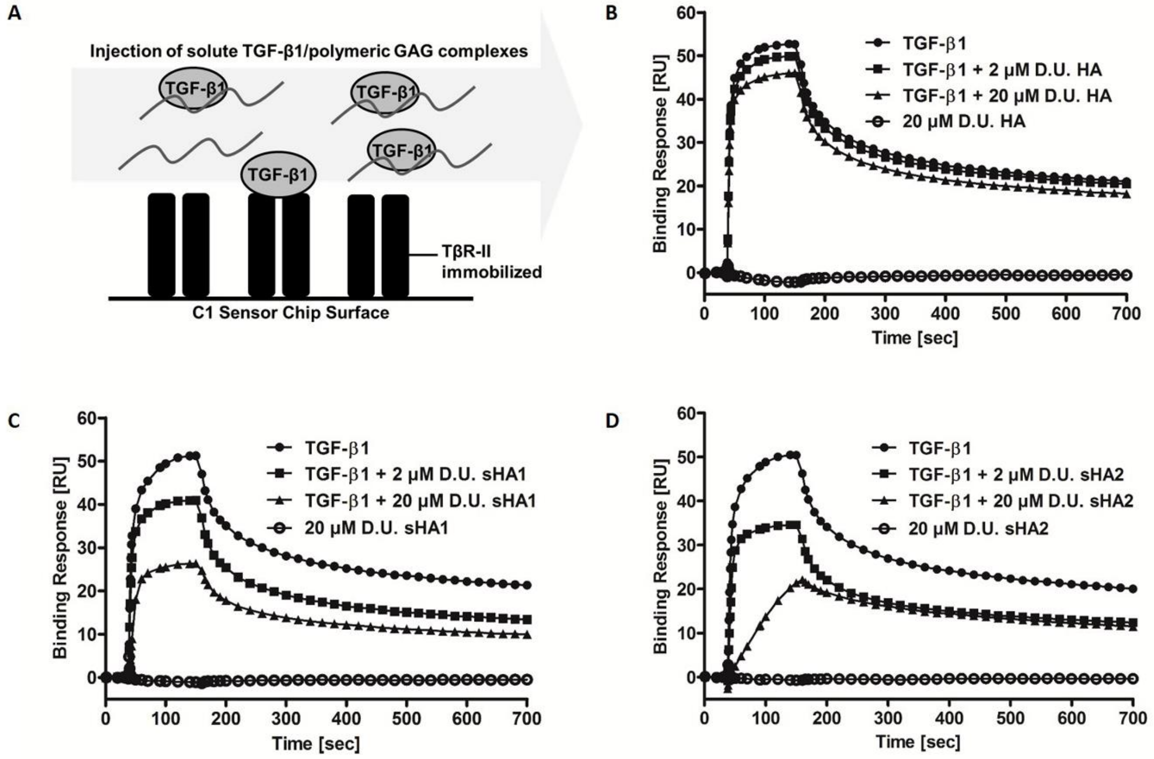

Supplementary Figure S1. Binding of TGF-β1 to immobilized TβR-II after pre-incubation with different GAG derivatives. (A) Schematic drawings of the experimental set-up. (B) Relative binding of 40 nM TGF-β1 to TβR-II alone and after pre-incubation with 2 and 20 µM D.U. of (B) HA, (C) sHA1 and (D) sHA2. One representative sensorgram out of three measurements is shown for each GAG derivative.

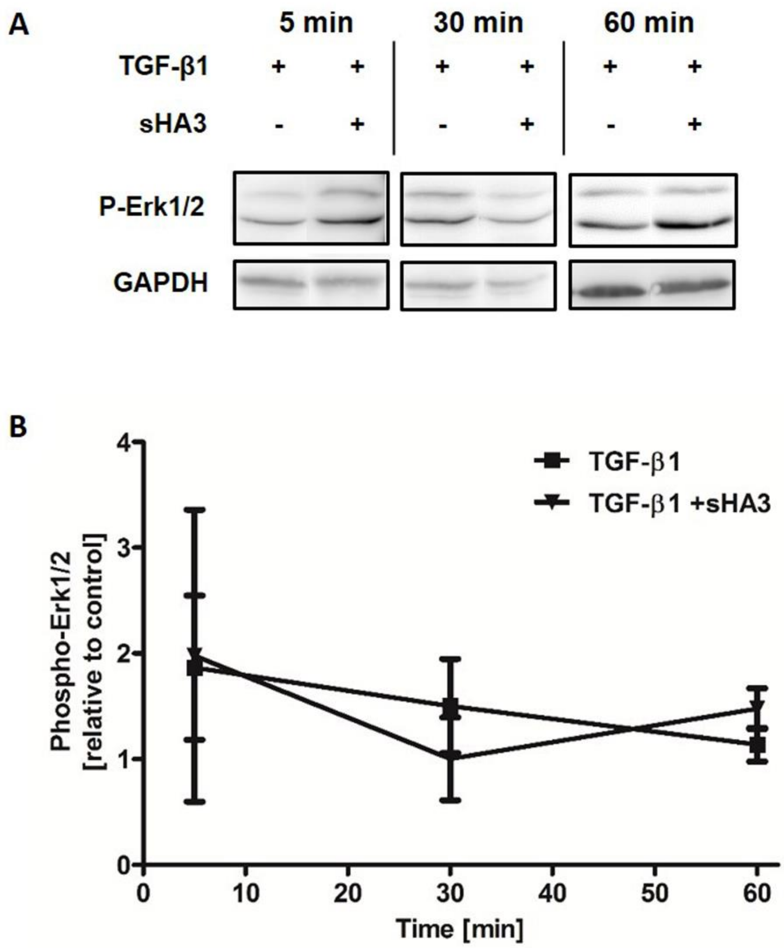

Supplementary Figure S2. Influence of sHA3 on the TGF-β1-mediated Erk1/2 phosphorylation. Hs27 fibroblast cells were stimulated with 10 ng/ml TGF-β1 (0.4 nM) alone or pre-formed complexes of TGF-β1 and 100 µg/ml sHA3 (130 µM D.U.) for the indicated time points. Cells were lysed and applied to Western Blot analyses using specific anti-phospho-Erk1/2 and GAPDH antibodies. (A) For every time point a representative blot is shown. The time course of TGF-β1-mediated phosphorylation in the presence and absence of sHA3 is plotted for (B) Erk1/2 phosphorylation relative to unstimulated cells.
